# Circular RNAs in obesity and related metabolic disorders: mechanistic insights and therapeutic perspectives

**DOI:** 10.3389/fgene.2026.1763596

**Published:** 2026-04-10

**Authors:** Daniela Bernal-Vázquez, Ignacio Bolaños-Fernández, Gabriel O. Salcedo-Rivas, Marcela Figueroa-Ramírez, Asim K. Duttaroy, Sujay Paul

**Affiliations:** 1 Tecnologico de Monterrey, School of Engineering and Sciences, Campus Querétaro, Querétaro, Mexico; 2 Department of Nutrition, Institute of Basic Medical Sciences, Faculty of Medicine, University of Oslo, Oslo, Norway

**Keywords:** biomarker, circular RNA, metabolic disorders, non-coding RNA, obesity, therapeutic

## Abstract

Obesity is a multifactorial and chronic metabolic disease strongly linked to type 2 diabetes mellitus, non-alcoholic fatty liver disease, cardiovascular complications, and several cancers. Despite advances in pharmacological and surgical interventions, long-term management remains suboptimal, emphasizing the need for new molecular targets. Circular RNAs (circRNAs), a unique class of covalently closed non-coding RNAs, have recently emerged as critical regulators of metabolic homeostasis. By acting as microRNA sponges, transcriptional modulators, and protein scaffolds, circRNAs participate in key processes such as adipogenesis, lipid metabolism, inflammation, and insulin signaling. Dysregulated circRNA expression has been implicated in obesity-induced β-cell dysfunction, hepatic steatosis, vascular remodeling, and tumor progression. Their high stability, evolutionary conservation, and tissue-specific expression further highlight their promise as minimally invasive biomarkers and therapeutic candidates for obesity-associated disorders. However, translation to clinical application remains limited due to methodological inconsistencies, lack of standardized quantification, and insufficient functional validation. Future integration of circRNA profiling with multi-omics and genome-editing approaches could accelerate the identification of mechanistic pathways and therapeutic targets, paving the way for precision medicine strategies in obesity and related metabolic diseases.

## 1 Introduction

Obesity has become a significant public health issue worldwide over the last 4 decades, with its prevalence almost doubling since 1980 (Salam et al., 2022). Clinically, it is defined as a complex, progressive, and relapsing chronic disease in which abnormal or excessive body fat impairs health and increases the risk of numerous long-term complications (Wharton et al., 2020). This epidemic results from a multifaceted interaction between environmental factors, genetic susceptibility, and individual behaviors (Omer, 2020). Fundamentally, the pathophysiology of obesity is driven by a disruption in energy homeostasis, a regulated process governed by hormonal and metabolic signals that control adipocyte differentiation, lipid metabolism, energy expenditure, and feeding behavior (Lustig et al., 2022). The metabolic dysregulation accompanying obesity significantly elevates the risk of developing type 2 diabetes mellitus (T2DM), with approximately 80% of T2DM patients being overweight or obese (Jin et al., 2023). Moreover, the accumulation of visceral white adipose tissue is a major contributor to cardiovascular diseases such as atherosclerosis and hypertension (Gutiérrez-cuevas et al., 2021). The liver is also severely affected, with non-alcoholic fatty liver disease affecting up to 90% of the obese population (Yang et al., 2022). Furthermore, obesity is now recognized as a major risk factor for several cancers, including endometrial, colorectal, and postmenopausal breast cancer (Santos and Sinha, 2021). These pathologies are largely driven by the dysfunction of adipose tissue, which undergoes cellular remodeling and changes in its secretome, promoting chronic inflammation or insulin resistance (Nowicka, 2024).

Current treatment strategies for obesity are multifaceted and personalized, often beginning with nutritional selection, diet restriction, and physical exercise, followed by cognitive behavioral strategies, pharmacological agents, or surgical procedures (Baker et al., 2022). These therapies aim to modulate key intracellular signaling pathways, including MAPK, PI3K/AKT, JAK/STAT, and Wnt/β-catenin pathways, that regulate critical processes such as appetite, adipogenesis, thermogenesis, and inflammatory responses (Wen et al., 2022). Pharmacotherapeutic options include glucagon-like peptide-1 (GLP-1) receptor agonists such as liraglutide, semaglutide, and tirzepatide, which have demonstrated promising efficacy in recent studies (Jędrasek et al., 2024). In cases of severe obesity, bariatric surgery remains the most effective intervention to efficiently lower body mass (Dragano et al., 2020). In addition, less invasive endoscopic procedures have emerged, especially for patients at higher surgical risk (Safdar et al., 2021). Furthermore, gut microbial modulation through fecal microbiota transplantation, probiotics, and prebiotics represents a potential strategy for improving host metabolism and weight regulation (Yang et al., 2022). Despite these diverse treatments, long-term strategies for managing obesity, including behavioral, pharmacological, and surgical interventions, offer modest success; treatment adherence remains low due to psychosocial, structural, and biological barriers (Bodepudi et al., 2024; Gonçalves et al., 2025). These limitations have intensified the search for novel molecular regulators that could support better diagnostic and therapeutic outcomes in obesity and its comorbidities. Among these, circular RNAs (circRNAs) have recently emerged as key modulators of adipogenesis, insulin sensitivity, and inflammatory signaling, and may represent promising targets for obesity therapeutics.

Non-coding RNAs (ncRNAs) are functional RNA molecules that are transcribed from the genome but not translated into proteins, and they participate in the regulation of several cellular processes, including transcription and RNA processing (Dandare et al., 2023). Among these ncRNAs, long non-coding RNAs (lncRNAs) and microRNAs are the most extensively studied. For instance, a growing body of evidence has demonstrated that are key regulators of the inflammatory pathways associated with obesity and its related chronic disorders (Wijesinghe et al., 2021), with some playing a pivotal role in regulating adipose tissue function and energy homeostasis (Dayal Aggarwal et al., 2024) and others contributing to lipid metabolism through epigenetic regulation (Kaimala et al., 2024). While research continues to uncover the complexities of these molecules, it is now known that IncRNAs are also involved in complex obesity metabolic syndromes by regulating lipid, cholesterol, and glucose metabolism (Ghafouri-Fard and Taheri, 2021). Similarly, several microRNAs (miRNAs) are also dysregulated in both obesity and rheumatoid arthritis, correlating positively with clinical measures and disease activity in these conditions (Auer et al., 2022); indeed, as adipose tissue is a major source of circulating miRNAs, their secreted profile can be completely altered during obesity (Heyn et al., 2020), and their dysregulated expression in inflammatory adipocytes is a key factor in obesity-associated inflammation (Kiran et al., 2021). Ultimately, the dysbalanced expression of various ncRNAs may cause numerous human disorders, such as cancer, cardiovascular, autoimmune, or infectious diseases (Niderla-Bielińska et al., 2023); while the less explored circular RNAs (circRNAs) represent a distinct class of single-stranded ncRNAs that form covalently closed loop structures through back-splicing, a process that imparts them with remarkable stability and resistance to exonuclease degradation (Li et al., 2020). They are considered as functional regulators with diverse biological roles, such as acting as microRNA (miRNA) sponges, protein decoys, and even templates for translation into peptides (Chen and Shan, 2021). Their expression is often tissue-specific and due to their sustained expression, conservation, and ability to modulate key biological processes via circRNA–miRNA and protein interactions, circRNAs are now recognized as important regulators in health and disease with growing potential for therapeutic applications (Zhao et al., 2022).

Given the limitations of current therapeutic approaches, there remains a persistent need to explore alternative molecular pathways that may enhance treatment efficacy and prevent disease progression. In this context, circRNAs could represent a promising molecular reservoir for obesity research. This scoping review aims to map and synthesize current evidence on the functional roles and therapeutic potential of circRNAs in obesity and obesity-associated disorders, highlighting key mechanisms, tissue-specific actions, assessing their promise as biomarkers or therapeutic tools, and identifying critical knowledge gaps to guide future research directions in the field.

## 2 Biogenesis and functional roles of circRNAs

**FIGURE 1:**
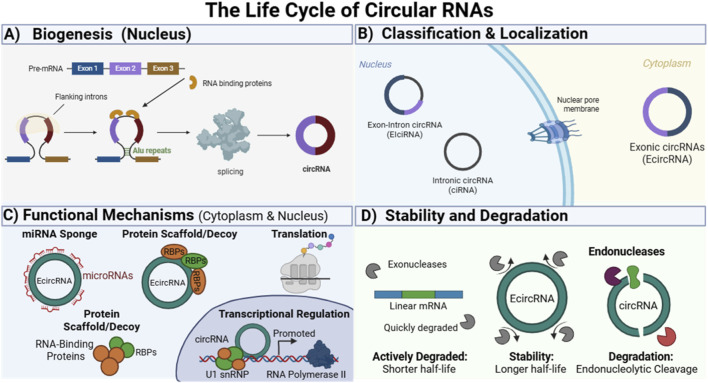
Life cycle, classification, functions, and stability of circular RNAs (circRNAs). **(A)** Biogenesis in the nucleus: circRNAs are uniquely formed through a backsplicing process catalyzed by the spliceosome, where flanking introns and complementary sequences facilitate exon circularization, often mediated by RNA-binding proteins (RBPs). **(B)** Classification and localization: circRNAs are categorized into exonic circRNAs (EcircRNAs), which primarily localize in the cytoplasm, and exon–intron circRNAs (EIciRNAs), which are predominantly retained in the nucleus to regulate gene expression. **(C)** Functional mechanisms: circRNAs participate in multiple regulatory processes, acting as microRNA (miRNA) sponges, protein scaffolds or decoys to modulate RBP activity, and in some cases, as templates for translation into functional peptides. **(D)** Stability and degradation: due to their covalently closed circular structure, which lacks free 3′ or 5′ ends, circRNAs are resistant to exonuclease-mediated degradation and exhibit longer half-lives than linear mRNAs; however, growing evidence suggests they can be degraded through specific, regulated pathways.

CircRNAs are uniquely formed through a backsplicing process, catalyzed by the spliceosome, where a downstream splice donor is covalently joined to an upstream splice acceptor (Stagsted et al., 2021). Backsplicing requires the coordinated involvement of spliceosome components and is tightly regulated by both cis-elements, such as inverted repeats in flanking introns, and trans-acting RNA-binding proteins (Wang et al., 2025). CircRNAs can be divided into three main subclasses: exonic circRNAs (EcircRNAs), composed exclusively of exonic sequences and generated by canonical backsplicing (Busa and Leung, 2021); exon–intron circRNAs (EIciRNAs), which retain introns, localize predominantly to the nucleus, and display strong tissue specificity (Zhong Y. et al., 2024); and interior circRNAs (i-circRNAs), which originate from internal regions of exons, introns, or intergenic transcripts via noncanonical pathways independent of canonical splicing signals (Liu et al., 2020a). An overview of circRNA biogenesis, classification, subcellular localization, functional mechanisms, and stability is summarized in Figure 1. A key driver of circularization is the presence of repetitive reverse complementary sequences, particularly Alu elements within flanking introns, which bring splice sites into proximity through stem-loop structures, thereby enhancing circularization efficiency (Meganck et al., 2021; Tao et al., 2021). In addition to sequence-driven pairing, RNA-binding proteins can promote circRNA biogenesis by binding to specific motifs within these intronic regions (Liang et al., 2021). The choice between producing linear or circular RNA is a regulated process, as depletion or inhibition of core spliceosome factors can shift gene output toward circRNA production (Xiao et al., 2020). Backsplicing can occur co-transcriptionally or post-transcriptionally, involving exon skipping, intron retention, and the use of cryptic splice sites (Srinivasan et al., 2025). Furthermore, many genes generate multiple circRNAs through alternative backsplice site selection, a process influenced by intron length and Alu element density across tissues (Mumtaz et al., 2020; Zhang et al., 2020). Additionally, circRNAs are found in extracellular fluids such as saliva, blood, and urine. They are structurally stable, with a half-life of approximately 48 h, significantly longer than that of mRNAs. Because exonic circRNAs (EcircRNAs) constitute the vast majority of known circRNAs and are the primary species detected in serum, they are of particular interest for clinical diagnosis; however, these serum EcircRNAs may be less stable due to the presence of circulating RNA nucleases (Zaiou, 2020). The stability and longer half-life of circRNAs, compared to their linear counterparts, are mainly due to their covalently closed circular structures, which lack free 3′ or 5′ ends, from which exonucleases typically degrade RNA molecules (Zhou et al., 2021). Although information regarding their degradation mechanisms is limited, growing evidence suggests the involvement of several pathways (Li et al., 2021; Ren et al., 2022).

CircRNAs play important roles in cellular physiology, particularly in transcriptional and post-transcriptional gene regulation. Although their primary functions remain unclear, various studies have shown that circRNAs can act as microRNA (miRNA) sponges, protein scaffolds, transcriptional regulators, and, in some cases, as templates for peptide translation (Venneri and Passantino, 2023). One of the most important mechanisms by which circular RNAs function is competing endogenous RNAs (ceRNAs), modulating the availability of microRNAs (miRNAs) for binding to their target mRNAs during translation. CircRNAs that harbor microRNA recognition elements (MREs) can bind to microRNAs (miRNAs) through stable base pairing. This interaction can suppress miRNA activity, thereby influencing the post-transcriptional regulation of their target genes (Zaiou, 2020). Another important mode of action of circRNAs involves their ability to bind proteins, thereby influencing protein localization, stability, and function. They can act as protein decoys in order to modulate the activity of RNA-binding proteins (RBPs) (Wu et al., 2022; Zhang et al., 2023). CircRNAs have also been linked to the regulation of cell death pathways, including autophagy and apoptosis, which are significantly associated with the development and progression of metabolic diseases. Recent studies highlight that circRNAs modulate autophagy through diverse mechanisms, altering transcription factor activity and influencing RNA methylation. These molecules can either promote or inhibit autophagy (Wang et al., 2025). It is well established that the majority of exonic circRNAs are localized in the cytoplasm. However, a specific fraction of these molecules, primarily exon-intron circRNAs (EIciRNAs) and circular intronic RNAs (ciRNAs), is predominantly retained in the nucleus. In this compartment, they play multifaceted roles in regulating gene expression beyond their classical function as miRNA sponges. Specifically, they can modulate transcription by recruiting RNA-binding proteins (RBPs) or chromatin remodeling complexes to gene promoters (Zhang et al., 2025a).

## 3 CircRNAs, adipose tissue, and obesity

CircRNAs exhibit distinct expression patterns, reflecting their diverse regulatory roles across different metabolic organs. This specificity has been observed in key tissues involved in energy balance, such as the liver, skeletal muscle and adipose tissue. In particular, distinct circRNAs have been observed between white adipose tissue (WAT), which primarily stores energy and brown adipose tissue (BAT), which dissipates energy through thermogenesis (Harsij et al., 2025). Obesity is characterized by excessive accumulation and dysfunctional remodeling of adipose tissue, which disrupts energy balance and contributes to systemic metabolic disease (Rahimi et al., 2023). Emerging studies indicate that circRNAs play crucial roles in this context by regulating adipocyte differentiation, lipid homeostasis, thermogenic activity, and inflammation (Shao et al., 2023). These functions are often mediated through post-transcriptional mechanisms, such as miRNA sponging or modulation of epigenetic regulators. The following subsections explore the specific roles of circRNAs in adipose tissue biology and their involvement in obesity-related metabolic dysfunctions.

### 3.1 CircRNAs in adipocyte development

Emerging studies underscore the essential role of circRNAs in controlling early stages of adipocyte differentiation and lipid accumulation (Li et al., 2022). Adipogenesis is a complex process by which stem cells differentiate into mature fat cells, driven by transcription factors such as peroxisome proliferator-activated receptor gamma (PPARγ) and CCAAT/enhancer-binding proteins (C/EBPs) that promote lipid storage (Mumtaz et al., 2020). In addition to transcriptional control, adipogenesis is regulated at the post-transcriptional level, where circRNAs can modulate gene expression by sequestering microRNAs (miRNAs) or interacting with RNA-binding proteins (Huang and Choo, 2023). In adipose tissue, circSAMD4A has been identified as a key modulator of preadipocyte differentiation. By sponging miR-138-5p, it releases the suppression of EZH2, an epigenetic regulator that silences target genes via H3K27 methylation. Notably, this circRNA is significantly upregulated in visceral fat of individuals with obesity, suggesting a role in obesity-related adipose tissue remodeling (Wen et al., 2025). Furthermore, experimental studies in humans and mice have shown that circSAMD4A overexpression promotes fat accumulation and adverse metabolic outcomes. In contrast, silencing this circRNA in obese mice leads to less weight gain and better insulin sensitivity and glucose tolerance, showing its important role in regulating fat cell formation (Liu et al., 2023b). Moreover, emerging evidence suggests that other, less characterized circRNAs may participate in adipogenesis via comparable regulatory networks, emphasizing their broad impact on adipose tissue remodeling.

### 3.2 CircRNAs in lipid homeostasis

**FIGURE 2:**
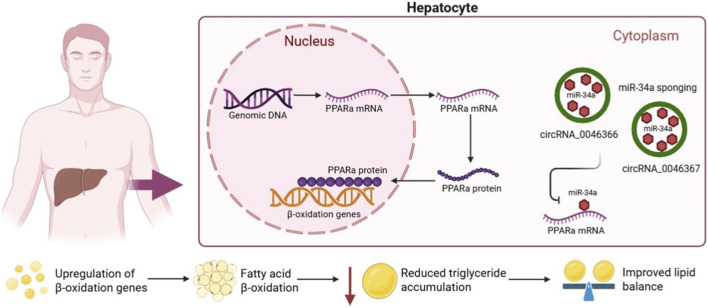
CircRNA-Mediated Regulation of Lipid Homeostasis in Hepatocytes. This diagram illustrates the mechanism by which circular RNAs, specifically circRNA_0046366 and circRNA_0046367, modulate lipid metabolism within a hepatocyte. In the cytoplasm, these circRNAs act as “sponges” by sequestering miR-34a. This prevents miR-34a from inhibiting the translation of PPARα mRNA. Consequently, there is an increased production of the PPARα protein, which then translocates to the nucleus and upregulates the expression of genes involved in fatty acid β-oxidation. The promotion of β-oxidation leads to a reduction in triglyceride accumulation, thereby improving overall lipid balance in the cell. *(Created with BioRender)*.

The regulation of lipid metabolism is fundamental for maintaining energy balance and metabolic health. Lipid homeostasis involves a dynamic balance between lipid storage (lipogenesis) and mobilization (lipolysis), processes essential for proper cellular function. The disruption of this balance not only alters the lipid composition but also can lead to the accumulation of toxic lipid species that interfere with protein stability and cell signaling (Vendruscolo, 2022). CircRNAs have emerged as key regulators of lipid metabolism, influencing the expression of genes involved in both synthesis and breakdown metabolism. In the liver, certain circRNAs regulate systemic lipid balance through post-transcriptional regulatory mechanisms like circRNA–miRNA–mRNA networks. For example, circRNA_0046366 and circRNA_0046367 have been shown to modulate fatty acid β-oxidation by binding to miR-34a, thereby lifting its inhibitory effect on Peroxisome Proliferator-Activated Receptor alpha (PPARα), a key nuclear receptor that controls lipid catabolism. This activation upregulates genes involved in β-oxidation, reducing triglyceride accumulation and improving lipid balance in hepatocytes (Guo et al., 2017; Małodobra-Mazur et al., 2024) (Figure 2). Such mechanisms point to circRNAs as promising therapeutic targets for metabolic liver diseases such as hepatic steatosis.

### 3.3 Thermogenic functions of circRNAs

The regulation of energy expenditure is an essential aspect of adipose tissue physiology and recent studies have discovered the role of circRNAs in thermogenesis. Thermogenesis is the process by which energy is dissipated as heat. In brown adipose tissue (BAT), this mechanism is essential for maintaining energy balance, particularly under cold stress, when metabolic activity is increased to produce heat. This process is tightly linked to overall energy metabolism and has become a key focus in understanding metabolic regulation (Zhang et al., 2025b). CircRNAs contribute to thermogenesis by regulating genes that control BAT development and activity. A robust example is circZEB1, which is significantly enriched in BAT. Mechanistically, circZEB1 acts as a molecular sponge for miR-326-3p, thereby promoting the differentiation of brown adipocytes and enhancing their thermogenic capacity. Experimental evidence demonstrates that this axis is crucial for maintaining thermal homeostasis and metabolic balance through the activation of thermogenic programs (Zhang, X. et al., 2022).

### 3.4 CircRNAs and chronic inflammation in adipose tissue

Chronic inflammation in adipose tissue is a key feature of obesity and plays a central role in the development of metabolic disorders such as insulin resistance and type 2 diabetes. A major contributor to this inflammatory state is the infiltration and activation of adipose tissue macrophages (ATMs), which adopt a proinflammatory phenotype, secrete cytokines such as TNF-α and IL-6 and accumulate lipids, thereby impairing adipocyte function (Zatterale et al., 2020; Makassy et al., 2025). Interestingly, circRNAs can modulate inflammatory signaling by influencing cytokine expression and the activity of inflammatory mediators (Joshi et al., 2025). For instance, circARF3 acts as a sponge for miR-103, a miRNA that targets tumor necrosis factor receptor-associated factor 3 (TRAF3), a signaling adaptor known for its role in modulating inflammatory pathways. By allowing TRAF3 expression, circARF3 indirectly limits the activation of stress-related kinases and the production of proinflammatory cytokines, possibly through enhanced mitochondrial autophagy (Liu et al., 2025). However, despite these examples, much remains to be understood about the broader roles of circRNAs in regulating adipose tissue inflammation and insulin resistance.

## 4 CircRNAs in obesity-associated metabolic disorders

**FIGURE 3:**
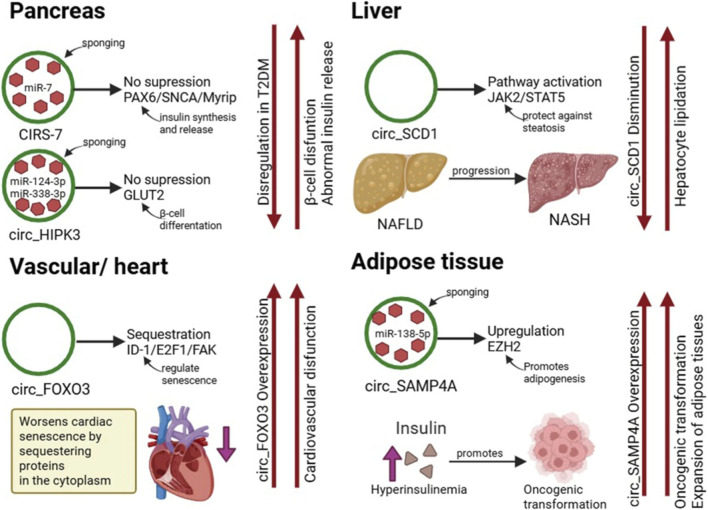
Tissue-specific circRNA mechanisms linking obesity to metabolic and cardiovascular pathology. Schematic summary of four organ-level circRNA axes illustrated in the manuscript. Top left (Pancreas): circHIPK3 and CDR1as (ciRS-7) act as competing endogenous RNAs that sequester miR-7, relieving repression of downstream targets such as PAX6 (and other β-cell genes) to modulate insulin production and β-cell function; dysregulation of these axes contributes to β-cell dysfunction and progression toward type 2 diabetes. Top right (Liver): Overview of the role of circSCD1 in the progression of non-alcoholic fatty liver disease (NAFLD) to non-alcoholic steatohepatitis (NASH). circSCD1 is downregulated in NAFLD/NASH, and its reduction is associated with disease progression, while its activity through JAK2/STAT5 is hepatoprotective. Bottom right (Adipose tissue/oncogenic linkage): circSAMD4A sponges miR-138-5p, releasing epigenetic regulators such as EZH2 (and other transcriptional effectors) to drive adipocyte differentiation, lipid accumulation and adipose expansion; chronic adipose dysfunction may additionally create a pro-tumorigenic microenvironment (annotated here as “oncogenic transformation”). Bottom left (Vascular/heart): circFOXO3 functions primarily via protein interactions and sequestration of stress-response factors (here annotated with reported effectors ID1, E2F1, FAK, HIF-1α), promoting cardiovascular dysfunction. Arrows denote direction of regulation; “↓” and “↑” indicate decreased and increased activity/expression, respectively. *(Created with BioRender)*.

The role of circRNAs in metabolic disorders has gained attention in recent years. Research has shown that circRNAs are significantly involved in T2DM, as they play key roles in regulating insulin production in pancreatic β-cells (Stoll et al., 2020), reducing obesity-induced β-cell apoptosis (Liu et al., 2023c), and contributing to impaired wound healing by mediating migration and proliferation of cells in diabetic foot ulcers (DFU) (Liang et al., 2023). Moreover, circRNAs also play relevant roles in other obesity-related disorders, such as the non-alcoholic fatty liver disease (NAFLD) (Liu et al., 2023a), hypertension (He et al., 2025), cardiovascular diseases (CVDs) (Yuan et al., 2024), and even obesity-linked cancer (Takenaka et al., 2023) (Figure 2). These findings point to the possibility of using circRNAs not only as biomarkers, but also as mediators of disease progression and, thus, potential therapeutic targets in a range of metabolic diseases. CircRNA-miRNA-protein regulatory networks contributing to obesity-associated metabolic diseases across major organs have been shown in Figure 3.

### 4.1 CircRNAs in insulin resistance and type 2 diabetes

Type 2 diabetes mellitus (T2DM) is an endocrine disorder marked by a or complete deficiency of insulin resulting from impaired function or loss of pancreatic β-cells (Qadir et al., 2022). In recent years, a growing body of research has revealed that circRNAs acting as miRNA sponges, such as ciRS-7 (also known as CDR1as) and circHIPK3, play a significant role in maintaining insulin secretion and β-cell function by sponging miR-7 and other miRNAs, and these interactions influence the expression of genes involved in insulin synthesis and release. Both ciRS7 and circHIPK3 are critical for sustaining insulin secretion and cell proliferation; In pancreatic β-cells, ciRS7 regulates insulin synthesis and secretion by sponging miR-7, thereby relieving its suppression of key target genes, including *PAX6*, *Myrip*, and *SNCA*, which are essential for β-cell differentiation, insulin transcription, and exocytosis (Xu et al., 2015). Similarly, circHIPK3 regulates several key β-cell genes, including *SLC2A2 (GLUT2)*, *AKT1*, and *MTPN*, primarily through interactions with miR-124-3p and miR-338-3p. Silencing circHIPK3 leads to reduced glucose-stimulated insulin secretion and impaired β-cell proliferation. Notably, both circRNAs are decreased in diabetic models, suggesting that their dysregulation may contribute to β-cell dysfunction and abnormal insulin homeostasis (Formichi et al., 2021; Fan et al., 2022). In contrast, recent evidence indicates that certain circRNAs are upregulated in the obesity/insulin resistance context as compensatory regulators of β-cell mass/function. For example, circGlis3 was upregulated in islets of obese/Lepr db/db mice, and when overexpressed, improved insulin secretion and decreased β-cell apoptosis via miR-124-3p sponging and SCOTIN binding (Liu et al., 2023a). Additionally, a recent high-throughput transcriptomic study involving peripheral blood samples from obese T2DM patients identified 442 differentially expressed circRNAs and constructed a circRNA–miRNA–mRNA network. Within this circulating profile, hsa_circ_0060614 was implicated via sponging miR-4668-3p to regulate MT2A, a gene involved in inflammation and oxidative stress pathways (Zhen et al., 2025a).

Analysis of serum samples from human patients revealed that hsa_circ_0056618 was upregulated in T2DM and inversely correlated with miR-206. This dysregulation was associated with increased PTPN-1 expression and decreased IRS protein levels; ROC analysis suggested that this circulating circRNA could serve as a diagnostic biomarker (AUC ∼0.93) (Abdelgwad et al., 2024). Current studies further indicate that dysregulated circRNAs not only affect β-cell function but also peripheral tissues involved in glucose homeostasis. In pancreatic β-cells, circRNAs can modulate autophagy, a process critical for maintaining cell function and insulin secretion. For example, rno_circRNA_008565 promotes β-cell autophagy by sponging miR-504 and modulating MAPK8 (JNK), while dysregulation of PI3K/AKT and MAPK pathways further impairs insulin signaling. In skeletal muscle, circHIPK3 may preserve insulin responsiveness by sequestering miR-29, which is upregulated in diabetic conditions and inhibits IRS-1/PI3K/AKT signaling, affecting glucose uptake and metabolism. Another circRNA, circAFF1, has also been shown to play a protective role in pancreatic islets; knocking it out increases the sensitivity of β cells to cytokine-induced apoptosis, emphasizing the contribution of circRNAs to the preservation of β-cell mass under inflammatory conditions (Kaur et al., 2022). These findings underscore circRNAs as critical modulators of both β-cell function and systemic insulin sensitivity through miRNA sponging and regulation of key signaling pathways (Samavarchi Tehrani et al., 2021).

### 4.2 CircRNAs in non-alcoholic fatty liver disease (NAFLD)

NAFLD is the most common liver disease worldwide, affecting around 30% of the population and is projected to continue growing due to increasing obesity rates (Cotter and Rinella, 2020). It is a chronic disease characterized by hepatic steatosis, defined as lipid accumulation in more than 5% of hepatocytes, and is recognized as an obesity-associated metabolic disorder that can progress from simple steatosis to non-alcoholic steatohepatitis (NASH), a more severe phenotype marked by lobular inflammation, hepatocyte ballooning, oxidative stress, and fibrogenesis. Over time, NASH can advance to cirrhosis, liver failure, or hepatocellular carcinoma, underscoring the importance of early intervention (Yepmo et al., 2022). There is a growing body of evidence that suggests that circRNAs are associated with the development and progression of NAFLD and NASH (Zeng et al., 2021). Indeed, a 2023 review by Zeng et al. has compiled the circRNA dysregulation landscape in NAFLD and NASH, highlighting multiple circRNAs associated with steatosis, inflammation, fibrosis and hepatocyte injury. Likewise, in a 2025 study, 59 differentially expressed circRNAs (DECs) were found between normal cells and NAFLD liver tissues, which shows the relationship between circRNA expressions and this hepatic disorder. This differential expression could be caused by m6A modifications, since high-confidence potential m6A modification sites were observed in 39 of the mentioned DECs (Zheng et al., 2025). Moreover, a 2024 study (Wang et al., 2024) indicates that aberrant expression of circSCD1 influences hepatocyte lipidation and may serve as a bridge between steatosis and progression to NASH. In line with the obesity-metabolic dysfunction axis, Fernández-Barrena et al. (2025) emphasize that circRNAs may not only reflect steatosis but also serve as biomarkers in Metabolic Dysfunction-Associated Steatotic Liver Disease (MASLD), thus offering a translational angle in the obesity context. Interestingly, in patients with both T2DM and NAFLD, a critical circRNA-mediated ceRNA network involving hsa_circ_0004535, miR-1827, and Caspase 8 (CASP8) has been identified. Mechanistically, hsa_circ_0004535 is downregulated in these patients, which fails to sponge miR-1827, leading to the subsequent repression of Caspase 8 (CASP8). The disruption of this axis contributes to metabolic dysfunction by promoting oxidative stress and aggravating hepatic lipid accumulation (Li et al., 2023).

### 4.3 CircRNAs in hypertension

Several miRNAs play an important role in regulating blood pressure and driving the development of hypertension by influencing vascular, renal, and other physiological pathways (He et al., 2025). Although research on circRNAs in essential hypertension is still limited, numerous miRNAs have already been strongly linked to the disease, underscoring the need for improved early diagnostic strategies. Most current evidence remains preclinical or from early clinical/observational studies. For instance, recent animal model studies involving the Renin-Angiotensin-Aldosterone System (RAAS)-driven hypertensive rat have identified circRNA candidates whose expression changes with antihypertensive treatment, suggesting a role for circRNAs in blood-pressure regulation via renal/RAAS pathways (Atchison, 2024). Similarly, Zheng et al. (2020) reported a significant association between the expression of hsa_circ_009102_5 in peripheral blood and hypertension. This circRNA was significantly upregulated in patients with essential hypertension compared to healthy controls, demonstrating diagnostic potential as part of a biomarker panel. Among the circRNAs investigated in blood samples, hsa_circ_0014243 has emerged as a particularly relevant molecule due to its multiple miRNA-binding sites, including a specific site for hsa-miR-10a-5p, suggesting its function as a molecular sponge (Zheng et al., 2020). Recent findings indicate that patients with hypertension exhibit reduced levels of hsa_circ_0014243 and upregulated levels of hsa-miR-10a-5p. Additionally, HSA_CIRC_0014243 has demonstrated strong diagnostic potential as a biomarker and appears to play an important role in the development and progression of hypertension, making it a promising therapeutic target (Zheng et al., 2018). Current studies further emphasize the potential of circRNAs as early diagnostic biomarkers in hypertension, owing to their cell- and tissue-specific expression, extracellular stability, and capacity to regulate miRNA and gene networks (Goina et al., 2024). While these human observational findings remain among the few in essential hypertension, translational work is now emerging, e.g., in hypertensive rodent models treated with RAAS inhibitors, circRNAs aligning with miRNA-regulated pathways have been identified, pointing toward mechanisms (and potential therapeutic targets) beyond mere biomarker associations (Atchison, 2024). Collectively, while literature remains nascent in essential hypertension, the convergence of biomarker, mechanistic and therapeutic-target data on circRNAs suggests that they may soon become part of the translational pipeline (diagnostic panels/therapeutic sponges) in hypertension management (Tarhriz et al., 2025).

### 4.4 CircRNAs in obesity associated cardiovascular diseases (CVDs)

In the cardiovascular system, numerous circRNAs have been identified and are involved in key processes of development and disease, often acting as miRNA sponges, interacting with RNA-binding proteins, or serving as biomarkers (Yuan et al., 2024). A major mechanism involves circRNAs functioning as “sponges” that absorb miRNAs, thereby positively regulating downstream proteins and playing important roles in the development of CVDs (Ju et al., 2021). Beyond acting as miRNA sponges, some circRNAs can serve as protein antagonists; for instance, circ-Foxo3 can worsen cardiac senescence by sequestering antisenescence and antistress proteins (such as ID-1, E2F1, FAK and HIF1α) in the cytoplasm. Specific circRNAs are also implicated in distinct pathologies, such as circANRIL, which confers a protective effect on atherosclerosis by promoting apoptosis and inhibiting proliferation in vascular smooth muscle cells and macrophages (Liu et al., 2021; Holdt et al., 2016). In the context of human coronary heart disease, a 2024 bioinformatics and validation study identified a panel of circRNA biomarkers via WGCNA/LASSO that could discriminate coronary artery disease patients from controls, underscoring translational potential (Zhong Q. et al., 2024). The stability of circRNAs (due to their closed-loop structure) and their disease/tissue-specific expression profiles make them attractive as circulating biomarkers. For example, hypoxia-induced PPARA-encoded circRNAs are upregulated in the blood of acute myocardial infarction patients (Huang et al., 2024), while circRNA CDR1as is upregulated during ischemia/reperfusion injury and contributes to the pathogenesis of myocardial infarction (Goina et al., 2024). Nonetheless, beyond biomarkers, therapeutic strategies (e.g., circRNA-sponges, antisense inhibition) are being explored, though delivery and specificity remain challenges (Yuan et al., 2024). In conclusion, it is important to note that most circRNA research in CVD remains at a pre-clinical stage or in small patient cohorts; large longitudinal studies, mechanism resolution (especially in obesity-mediated vascular/adipose-cardiac crosstalk), and *in vivo* therapeutic delivery are still nascent. Integrating circRNA data with obesity-mediated inflammation, adipose tissue dysfunction and metabolic syndrome may be a fruitful next step (Mei et al., 2024).

**FIGURE 4:**
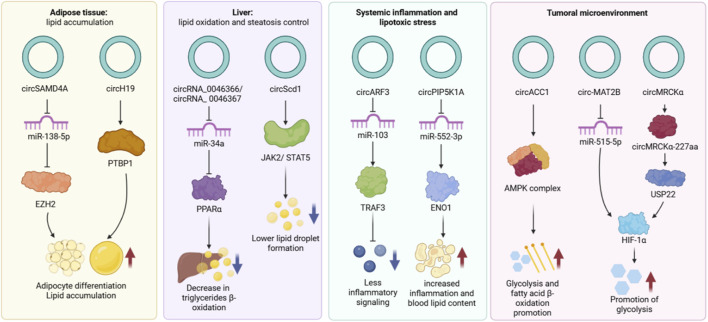
Multi-organ regulatory networks of circular RNAs in the control of adipocyte differentiation, hepatic lipid metabolism, and systemic inflammatory stress. This schematic illustrates the stage-specific molecular mechanisms by which circRNAs orchestrate metabolic homeostasis across multiple physiological and pathological contexts. In the Adipose Tissue stage, circSAMD4A acts as a competitive endogenous RNA by sequestering miR-138-5p to upregulate EZH2, while circH19 interacts with PTBP1; both pathways cooperatively promote adipocyte differentiation and lipid accumulation. In the Liver stage, circRNA_0046366, circRNA_0046367, and circScd1 function as critical regulators of lipid oxidation and steatosis control; the former axis inhibits miR-34a to increase PPARα-mediated β-oxidation and reduce triglycerides, while the latter activates JAK2/STAT5 signaling to limit lipid droplet formation. In the Systemic Inflammation and Lipotoxic Stress stage, circARF3 modulates inflammatory signaling by inhibiting the miR-103/TRAF3 axis, whereas circPIP5K1A sequesters miR-552-3p to regulate ENO1, leading to increased inflammation and raised blood lipid content. Finally, in the Tumoral Microenvironment, circRNAs participate in obesity-associated metabolic reprogramming: circACC1 activates the AMPK complex to promote glycolysis and fatty-acid β-oxidation, circ-MAT2B modulates the miR-515-5p/HIF-1α axis to enhance glycolytic metabolism under hypoxic conditions, and circMRCKα regulates USP22 through circMRCKα-227aa, contributing to HIF-1α stabilization and glycolysis promotion. Together, these networks illustrate how circRNA-mediated regulation integrates metabolic homeostasis with inflammation-driven and hypoxia-associated metabolic remodeling in cancer-related contexts *(Created with BioRender).*

A global overview of the multi-organ circRNA regulatory networks involved in metabolic homeostasis is presented in Figure 4.

### 4.5 CircRNAs and obesity-linked cancer

Obesity is a complex and multifactorial disease that contributes to the development of various disorders, including cancer. It has been considered a factor that favors tumorigenesis by promoting chronic inflammation, insulin resistance, and a proinflammatory tumor microenvironment. However, recent studies have described the so-called “obesity paradox” in which it is reflected as a risk factor for the development of cancer and a protective phenotype for established cancers. For instance, transcriptomic profiling in obesity-related malignancies (such as endometrial carcinoma in obese women) has revealed extensive differential circRNA expression, with hundreds of circRNAs altered in obese cancer patients vs. non-obese or adjacent normal tissue (Takenaka et al., 2023). Interestingly, patients with mild obesity may present greater sensitivity to antitumor therapies, particularly immune checkpoint inhibitors (Assumpção et al., 2022); this finding highlights the complexity of the obesity-cancer relationship and suggests that circRNAs could play a modulatory role in both scenarios.

Certain circRNAs serve as key regulators connecting metabolic reprogramming and tumorigenesis. This is the case with circSAMD4A, which has been shown to modulate adipocyte differentiation by acting as a “sponge” for miR-138-5p, promoting the upregulation of EZH2, thus contributing to the expansion of adipose tissues in obesity (Liu et al., 2020b). Chronic adipose dysfunction promotes oncogenic transformation through increased lipid availability, hyperinsulinemia and systemic inflammation. Additionally, mounting evidence indicates that in obesity-linked cancers, circRNAs may function not only via adipogenesis pathways but also by modulating tumor metabolism (e.g., glycolysis), microenvironmental inflammation, or lipid availability to tumor cells (Hsu et al., 2024).

In line with these observations, recent evidence identifies specific circRNAs that directly reprogram metabolic pathways to fuel tumor growth. For instance, circACC1 exhibits versatile oncogenic mechanisms depending on the tumor context. In colorectal cancer, it acts as a critical metabolic regulator. Under stress conditions, c-Jun unregulates circACCI, which then acts as a scaffold to stabilize the AMPK holoenzyme complex. This reaction promotes fatty acid β-oxidation and glycolysis, providing the metabolic flexibility required for tumor cell survival (Li et al., 2019). Similarly, in gastric cancer, circ-MAT2B facilitates metabolic reprogramming by sponging miR-515-5p to upregulate HIF-1α, thereby promoting glycolysis and accelerating malignant progression even under hypoxic conditions (Liu et al., 2020c). This mechanism is relevant in obesity, as the expansion of adipose tissue creates a systemic hypoxic environment that further amplifies HIF-1α signaling, favoring tumorigenesis in metabolic cancers (Michailidou, 2019). Furthermore, the interplay between inflammation and metabolism is demonstrated by cicrcMRCKa in hepatocellular carcinoma. As demonstrated by Yu et al. (2024), this circRNA is induced by tumor-associated macrophages (TAMs), which are abundant in obese liver and encodes a peptide that promotes glycolysis, illustrating how TAMs induce inflammation and concurrently promote tumor glycolysis, thereby linking immune signaling to metabolic reprogramming.

Further, circRNAs are increasingly recognized as regulators of the tumor microenvironment. For example, a 2024 review highlights that circRNAs can modulate immune cell infiltration, angiogenesis, and extracellular matrix remodeling, all of which are influenced by the obese microenvironment of excess adiposity and chronic inflammation (Botwe et al., 2024). Given their remarkable stability in body fluids and tissue-specific expression, circRNAs represent promising non-invasive biomarkers in obesity-linked cancers and potential therapeutic targets, including approaches based on antisense inhibition, circRNAs sponges, or synthetic circRNAs delivery. Unfortunately, most studies to date remain correlative or descriptive; functional validation in obesity-driven tumor models, the impact of weight reduction/bariatric surgery on circRNAs profiles in tumors, and the integration of circRNAs signatures with metabolic phenotyping are still largely missing.

The main circRNAs acting as miRNA sponges in obesity and related metabolic disorders are summarized in Table 1 and CircRNAs acting as RBP decoys and their associated pathways are summarized in Table 2.

**TABLE 1:** Functional Roles and Molecular Mechanisms of Circular RNAs acting as miRNA sponges.

CircRNA	Targeted miRNA	Affected pathway	Sample source	Type of expression	Biological function	Author
circHIPK3	miR-20b-5p	VEGFA	Human umbilical vein endothelial cells	Upregulated	Enhance wound healing by promoting angiogenesis, cell proliferation and migration in diabetic foot ulcers	Liang et al. (2023)
	miR-192-5p	FOXO1	Cultured HepG2 and Huh7 cells	Upregulated	Contributes to hyperglycemia and insulin resistance	Cai et al. (2020)
	miR-185	Cyclin D1 TGF-β1 PCNA	Isolated rat kidney and cultured rat mesangial cells	Upregulated	Exacerbates diabetic nephropathy by regulating cell proliferation	Liu et al. (2021)
CDR1as	miR-7	MST1 Hippo pathway	Cultured neonatal mouse cardiomyocytes	Upregulated	Induces apoptosis in diabetic cardiomyopathy	Shao et al. (2022)
		PAX6 Myrip SNCA	Cultured MIN6 cells and isolated mouse islets	Upregulated	It regulates the synthesis and secretion of insulin in pancreatic β-cells	Xu et al. (2015)
circGlis3	miR-124-3p	NeuroD1 Creb1	Cultured MIN6 cells and isolated mouse islet and exocrine glands; human serum and islets	Upregulated	Promotes insulin expression Reduces obesity-induced apoptosis in β-cells	Liu et al. (2023a)
circRNA_0084043	miR-140-3p	TNF-α IL-6 Cox-2	Cultured ARPE-19 cells	Upregulated	Prompts progression of diabetic retinopathy	Li et al. (2020)
circ_0002570	miR-1243	AMOT	Retinal proliferative fibrovascular membranes, cultured hRMECs	Upregulated	Inhibits angiogenesis, cell proliferation and migration in Diabetic retinopathy	Liu et al. (2020a)
circPIP5K1A	miR-552-3p	ENO1	Rat peripheral blood and pancreas samples	Upregulated	Increases insulin resistance and lipid metabolism disorder, exacerbates inflammation and induces histopathological changes in pancreas	Song et al. (2024)
circAFF1	Not reported	Not reported	Human and rat pancreatic islets	Downregulated	Protective function reduces the sensitivity of β-cells to cytokine-induced apoptosis	Stoll et al. (2018), Kaur et al. (2022)
hsa_circ_0044623	hsa-mir-129-5p	MYLK3	Human peripheral blood samples	Upregulated	Prevents disordered sarcomere structure, oxidative stress and reduced supply of oxygen in fatigue-type T2DM	Zhen et al. (2025b)
hsa_circ_0002622	hsa-mir-200b-3p	RAB21	Human peripheral blood samples, mouse pancreatic, liver, and skeletal muscle tissues	Upregulated	Reduces insulin resistance by regulating GLUT1 levels on the cell surface in fatigue-type T2DM	Zhen et al. (2025a)
hsa_circ_0060614	miR-4668-3p	MT2A	Venous blood	Upregulated	Involved in the regulation of inflammation/oxidative stress pathways (identified in circRNA-miRNA-mRNA network in patients with T2DM)	Zhen et al. (2025b)
Rno_circRNA_008565	miR-504	MAPK8 (JNK)PI3K/AKT MAPK	Isolated rat islets β-cells	Downregulated	It promotes autophagy of β-cells, dysregulation of the PI3K/AKT and MAPK pathways impair insulin signaling	Samavarchi-Tehrani et al. (2021) Bai et al. (2019)
circ_0068087	miR-580-3p	PAQR3	Human serum, cultured HK2 cells	Upregulated	Induces apoptosis, inflammation and oxidative stress in kidney cells	Liu et al. (2023b)
circSAMD4A	miR-138-5p	EZH2	Human adipose tissue samples, cultured human preadipocytes, mouse subcutaneous and visceral adipose tissue	Upregulated	Promotes preadipocyte differentiation	Liu et al. (2020b)
circRNA_0046366	miR-34a	PPARα	Cultured HepG2 cells	Downregulated	Upregulates PPARα, which in turn leads to the transcription of genes associated with lipid metabolism, alleviating steatosis	Guo et al. (2018), Wu et al. (2022)
circRNA_0046367	miR-34a	PPARα CPT2 ACBD3	Human liver tissues	Downregulated	Upregulates PPARα, which in turn leads to the transcription of genes associated with lipid metabolism, alleviating steatosis	Guo et al. (2018), Wu et al. (2022)
circZEB1	miR-326–3p	UCP1 PPARγ FABP4 FASN ELOVL3 adiponectin	Goat brown preadipocyte	Upregulated	Promotes brown adipocytes differentiation and thermogenesis	Zhang et al. (2022)
circARF3	miR-103	TRAF3	Primary preadipocyte culture, mouse adipose tissue	Downregulated	Promotes mitophagy, alleviating adipose inflammation	Liu et al. (2025) Zhang et al. (2018)
circRNA0056618	miR-206	PTPN-1 IRS protein	Peripheral venous blood	Upregulated	Overexpressed in T2DM, it correlates inversely with miR-206; increased PTPN-1 leads to decreased IRS protein	Abdelgwad et al. (2024)
HSA-circRNA-9102-5	hsa-miR-150-5p	PTX3	Peripheral blood	Upregulated	Significantly upregulated in patients with essential hypertension	Zheng et al. (2020)
HSA_circ_0014243	HSA-MIR-10A-5P	NF-κB MAPK7 β-transducin-repeat-containing gene	Blood	Upregulated	Reduced levels in patients with essential hypertension suggest a function as a molecular sponge and a potential biomarker in the development and progression of hypertension	Zheng et al.(2018)
​	miR-552-3p	ENO1	Rat peripheral blood and pancreatic tissues	Upregulated	Promotes inflammation, insulin resistance and increases lipid content	Song et al. (2024)

**TABLE 2:** Functional Roles and Molecular Mechanisms of Circular RNAs acting as RBP decoys.

CircRNA	Targeted RBP	Affected pathway	Tissue	Type of dysregulation	Biological function	Author
ci-INS	TDP-43	Cacna1d Syt4 Syt7 Pclo	Mouse, rat and human islets	Downregulated	Promotes β-cell function, induces insulin synthesis	Stoll et al. (2020)
circSCD1	JAK2 STAT5	JAK2/STAT5	Mouse liver, cultured AML-12 cells	Downregulated	Influences the clearance of hepatocytes and can serve as a bridge in the progression from steatosis to NASH	Wang et al. (2024)
circANRIL	PES1	Pre-rRNA maturation (p53)	Cultured HEK-293, MonoMac, iPSC Primary arterial SMC and FB cells	Downregulated	Protective effect in atherosclerosis by promoting apoptosis and inhibiting cell proliferation	Liu et al. (2021)
Hsa_circ_0131202 (DICAR)	VCP	Med12	Human peripheral blood, mouse heart tissue	Downregulated	Inhibits pyroptosis in diabetic cardiomyopathy	Yuan et al. (2023)
circ-foxo3	CDK2 P21	Cyclin E/CDK2 complex	Cultured MEF, NIH3T3, 4T07 and 4T1 cells	Upregulated	Worsens cardiac senescence by sequestering proteins in the cytoplasm	Du et al. (2016), Li et al. (2020)
	KAT7	HMGB1	Cultured H9c2 cells, rat heart tissue	Downregulated	Amelioration of myocardial ischemia or reperfusion injury, by reduced myocardial infarction size and improved cardiac function, resulting from the suppression of excessive autophagy, cardiomyocyte apoptosis, and inflammatory response	Sun et al. (2021)
circH19	PTBP1	SREBP1	Primary human ADSCs	Upregulated	Regulation of adipogenesis characterized by the acceleration of adipocyte differentiation where the downregulation of circH19 leads to increased formation of lipid droplets	Zhu et al. (2020)
circGlis3	GMEB1 MIB2	HSP27	Venous blood, mouse islets, cultured MIN6 cells	Upregulated	Reduced viability, migration and angiogenesis in islet endothelial cells	Xiong et al. (2022)

## 5 Current challenges and future directions

The potential of circRNAs as therapeutic targets or diagnostic markers in obesity-related diseases is remarkable. CircRNAs have been shown to significantly influence the development and progression of obesity and its associated disorders by regulating adipogenesis, inflammation, insulin resistance, lipid metabolism, and adipocyte differentiation (Liu et al., 2020d; Hamdy et al., 2024). Moreover, emerging evidence suggests that circRNAs can modulate chromatin remodeling and DNA methylation via interaction with epigenetic regulators such as EZH2 and DNMT1, thereby influencing adipocyte differentiation and inflammatory gene expression. Furthermore, their superior stability compared to linear RNAs, along with tissue- and disease-specific expression patterns, make circRNAs promising candidates for the early detection of obesity-related disorders. In particular, circRNAs directly involved in disease progression hold great promise as reliable clinical biomarkers. CircRNAs packaged into extracellular vesicles or exosomes are increasingly recognized as stable carriers of metabolic information, offering minimally invasive routes for biomarker discovery. However, their clinical validation remains limited. Major obstacles include the lack of standardized methods for quantifying circRNA expression, inconsistent adherence to the Minimum Information for Publication of Quantitative Real-Time PCR Experiments (MIQE) guidelines, ambiguous biomarker identification, the use of inadequate controls, and reliance on poorly described single retrospective cohort studies (O’Leary et al., 2025). Integrative multi-omics approaches combining circRNA, miRNA, proteomic, and metabolomic profiling are beginning to uncover coordinated regulatory networks in obesity and its metabolic complications. These system-level analyses may enable the identification of circRNA signatures predictive of disease progression or therapeutic response. Direct modulation of circRNA expressions, either upregulation or downregulation, as a therapeutic strategy for obesity-related diseases, has so far been minimally explored. Nonetheless, emerging approaches such as antisense oligonucleotide-mediated knockdown and CRISPR/Cas-based editing have become powerful tools for functional analysis of circRNAs. These technologies hold substantial promises for therapeutic development, pending optimization of delivery systems and a deeper understanding of potential off-target effects (Aquino-Jarquin, 2024).

## 6 Conclusion

Circular RNAs have emerged as crucial regulators of metabolic balance, influencing adipogenesis, lipid metabolism, insulin signaling, and inflammatory responses central to obesity and its comorbidities. Their tissue-specific expression, evolutionary conservation, and stability position them as attractive candidates for biomarker discovery and therapeutic development. However, translating these findings into clinical practice remains challenging due to technical variability, limited mechanistic insight, and incomplete functional validation *in vivo*. Advancing the field will require standardized circRNA quantification methods, integration with transcriptomic and proteomic datasets, and robust experimental models that account for the complexity of metabolic regulation. Eventually, elucidating the mechanistic networks governed by circRNAs could enable their application as diagnostic tools and therapeutic targets, opening new frontiers in precision medicine for obesity and obesity-associated disorders. Nonetheless, bridging preclinical insights to human translation will require standardized biobanking, longitudinal profiling, and validation in large multicenter cohorts to establish circRNAs as actionable biomarkers or therapeutic targets.
